# Anatomical and component rotational mismatches negatively affect postoperative outcomes in total knee arthroplasty

**DOI:** 10.1002/jeo2.70415

**Published:** 2025-09-09

**Authors:** Kohei Kawaguchi, Ryota Yamagami, Kenichi Kono, Junfeng Zhang, Shuji Taketomi, Hiroshi Inui, Sakae Tanaka

**Affiliations:** ^1^ Department of Orthopaedic Surgery The University of Tokyo Tokyo Japan; ^2^ Department of surgery Auckland University Auckland New Zealand; ^3^ Saitama Medical Center Saitama Medical University Saitama Japan

**Keywords:** anatomical rotational mismatch, clinical outcome, component rotational mismatch, rotational mismatch, total knee arthroplasty

## Abstract

**Purpose:**

Anatomical rotational mismatch (ARM) refers to postoperative malrotation between the femur and tibia, and component rotational mismatch (CRM) refers to malrotation between the femoral and tibial components in total knee arthroplasty (TKA). This study aimed to quantify ARM and CRM and assess their individual and combined effects on postoperative outcomes.

**Methods:**

This retrospective study analysed 224 knees that underwent primary TKA. Postoperative axial rotational angles between the femoral and tibial components (component rotational angle [CRA]) and between the femur and tibia (anatomical rotational angle [ARA]) were measured using computed tomography. Internal tibial or tibial component rotation relative to the femur or femoral component was assigned a positive value. Rotational mismatch was defined as CRA and ARA over ±10°. Clinical outcomes were evaluated using the Knee Injury and Osteoarthritis Outcome Score (KOOS) 1 year postoperatively. Hierarchical cluster analysis categorised knees into two groups based on CRA and ARA (Groups 1 and 2).

**Results:**

The mean ± standard deviation (SD) postoperative ARA was 5.2° ± 5.3°, with ARM present in 16.1% of cases (36 knees). Knees with ARM showed significantly worse improvement in the KOOS pain subscale than those without ARM (*p* = 0.02). Postoperative CRA was 1.5° ± 4.5°, with CRM observed in 2.7% of cases (6 knees), but CRM alone did not significantly affect postoperative outcomes. Cluster analysis identified two groups (Group 1: 185 knees; Group 2: 39 knees), with Group 2 exhibiting greater CRAs and ARAs compared to Group 1 (both *p* < 0.01). Group 2 also had significantly worse KOOS pain and activities of daily living improvement relative to Group 1 (*p* < 0.01, 0.04).

**Conclusions:**

Both CRM and ARM were observed following TKA. ARM negatively impacted postoperative outcomes, and the combined presence of CRM and ARM further worsened clinical results.

**Level of Evidence:**

Level IV.

**Abbreviations**
ADL: activities of daily living
AP: anteroposterior
ARA: anatomical rotational angle
ARM: anatomical rotational mismatch
BCS: bicruciate‐substitute
CRA: component rotational angle
CRM: component rotational mismatch
CT: computed tomography
KOOS: Knee Injury and Osteoarthritis Outcome Score
QOL: quality of life
SEA: surgical epicondylar axis
TKA: total knee arthroplasty

## INTRODUCTION

Approximately 20% of patients express dissatisfaction over the total knee arthroplasty (TKA) outcomes [5, 11]. Among reported various contributing factors [8, 10, 14, 22], femorotibial rotational malalignment is an unsolved contributing factor to dissatisfaction [40]. The increasing use of perioperative computed tomography (CT) has brought greater attention to axial femorotibial rotational alignment mismatch at knee extension. Postoperative rotational mismatches fall into two categories: mismatch between bones and mismatch between components [17, 27, 34]. ‘Anatomical’ rotational mismatch (ARM) refers to misalignment between the femoral and tibial rotational bone axes, whereas ‘component’ rotational mismatch (CRM) refers to misalignment between the femoral and tibial component axes.

Studies have suggested that femorotibial bone alignment may change after TKA [27, 37], sometimes leading to ARM. Postoperative ARM, defined as an anatomical femorotibial rotational angle >10°, occurs in 13%–29% of fixed‐bearing TKAs and 8%–13% of mobile‐bearing TKAs [18, 35, 37]. ARM has been identified as a clinical risk factor for poor postoperative functional outcomes in fixed‐bearing TKA [15, 34]. Anatomical rotational knee alignment also markedly affects patellar kinematics and patellofemoral joint alignment [3, 4, 21, 24, 38]. The rotational alignment of each component is a key risk factor for postoperative ARM in fixed‐bearing TKA [27, 34], emphasising the need for precise component positioning to minimise ARM. However, research on ARM is limited, and its clinical significance remains debated.

Postoperative CRM has been linked to poor clinical outcomes and TKA revision [13, 25], knee pain [23, 30] and increased patellofemoral joint pressure [36]. A cadaveric study demonstrated that the rotational alignment between the tibial and femoral components significantly impacts post‐TKA femorotibial rotational kinematics, with greater CRM leading to increased kinematic conflict compared to natural knee kinematics [26]. Like ARM, CRM is induced by femoral and tibial component malalignment [17], highlighting the need for precise rotational component alignment. Although ARM and CRM can occur together and interact, no study has evaluated them simultaneously in the same cohort. Moreover, ARM and CRM's individual and combined effects on postoperative outcomes remain unknown. This study aimed to evaluate ARM and CRM within the same cohort and investigate their individual and combined effects on clinical outcomes following TKA.

## PATIENTS AND METHODS

### Study design

This study was prospectively performed at a single institute and retrospectively reviewed. Between June 2018 and December 2021, 254 consecutive primary bicruciate‐substitute (BCS) TKAs (Journey II BCS; Smith & Nephew, Memphis, TN, USA) were performed using image‐free navigation devices (Precision N; Stryker Orthopedics, Mahwah, NJ, USA) to treat primary knee osteoarthritis. In total, 224 BCS TKA knees were included in this study. Among 254 knees with BCS TKA, we excluded 21 knees with a flexion contracture greater than 20° to ensure accurate assessment of knee extension rotational angles, as well as those with a postoperative knee extension angle change exceeding 10° relative to the preoperative angle. Additionally, nine knees lacking perioperative CT scans or clinical outcome data were excluded. Table 1 presents the patients' preoperative demographic data and characteristics.

**Table 1:** Preoperative patients' demographic and clinical data.

Knees (patients)	224 (182)
Age (years old)	72.8 ± 7.7
Female:Male	186:38
Right:Left	110:114
Height (m)	1.54 ± 0.1
Weight (kg)	62.8 ± 13.2
BMI (kg/m^2^)	26.4 ± 4.3
Knee extension angle (°)	−8.5 ± 6.6
Knee flexion angle (°)	118.7 ± 15.4
Femorotibial angle (°)	184.1 ± 6.1

*Note*: Data are shown average ± standard deviation.

Abbreviation: BMI, body mass index.

### Radiographic evaluation

Postoperative femorotibial anatomical and component rotational alignment were evaluated using a validated method [18, 19, 34] with CT scans and the Zed Knee System (LEXI, Tokyo, Japan) for 3D analysis. CT scans, with a slice thickness of 1 mm, were obtained from the hip to the ankle joint preoperatively and postoperatively (at the final preoperative clinical visit and 2 weeks after surgery, respectively). Scans were then imported into the Zed Knee System for analysis. This software, validated for CT‐based 3D preoperative planning and postoperative evaluation in TKA [18, 34], identified reference points on CT images to align the preoperative femorotibial axis' coordinates.

For the femur, reference points included the centres of the femoral head, the medial sulcus, and the lateral epicondyle on preoperative CT scans. For the tibia, reference points comprised the centres of the tibial anterior cruciate ligament attachment and the medial and lateral malleolus. Additionally, the femoral surgical epicondylar axis (SEA) and the anatomical tibial AP (anteroposterior) axis, known as ‘Akagi's line,’ which extends from the middle of the posterior cruciate ligament to the medial edge of the patellar tendon attachment [2], were defined as rotational axes on preoperative CT scans. Preoperative and postoperative CT images were automatically fused by aligning bone surfaces, allowing for the projection of reference points and rotational axes onto postoperative images.

**Figure 1:**
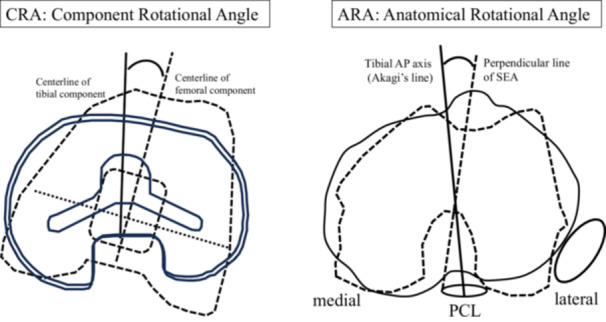
Schema of the three‐dimensional computed tomography measurement of femorotibial component rotational angle (CRA) and anatomical rotational angle (ARA). Components rotational angle (CRA) was the angle between the centre lines of femoral and tibial component, and anatomical rotational angle (ARA) was the angle between Akagi's line as tibial anteroposterior (AP) axis and the perpendicular line of surgical epicondylar axis (SEA) as femoral AP axis. SEA was drawn from the sulcus of the medial epicondyle to the lateral epicondyle and Akagi's line was drawn from the center of the attachment of posterior cruciate ligament to the medial border of the patella tendon. PCL, posterior cruciate ligament.

The femorotibial anatomical rotational angle (ARA) was defined as the angle of the anatomical tibial AP axis (Akagi's line) relative to the line perpendicular to the femoral SEA on CT scans, representing ‘anatomical rotational mismatch’ (Figure 1). On the other hand, the femorotibial component rotational angle (CRA) was defined as the angle of the tibial component's AP axis relative to the femoral component's AP axis, representing ‘component rotational mismatch’ (Figure 1). The mismatch threshold for both ARA and CRA was defined as two values: ±5° and ±10° according to previous studies [13, 16, 27, 33]. The rotational angles of the tibial component from Akagi's line and the femoral component from SEA were measured. Coronal and sagittal component alignments were evaluated using angles between the components and mechanical axes in both planes. Whole‐leg coronal plane radiographs under weight‐bearing conditions were also obtained to assess dynamic coronal limb alignment as a preoperative coronal femorotibial angle.

### Surgical procedure

All patients underwent an anterior skin incision and a medical parapatellar surgical approach without patellar eversion. The distal femur and proximal tibia were resected using a navigation system (Precision N; Stryker Orthopedics, Mahwah, NJ, USA). The femur was aligned at 90° to the mechanical axis in the frontal plane, with 4° of flexion in the sagittal plane. The tibia was also aligned at 90° to the mechanical axis in the frontal plane, with a 3° posterior slope in the sagittal plane. Our navigation was an image‐free navigation, therefore both rotational alignments were decided anatomically and mechanically. Femoral component rotation was set parallel to the SEA based on the posterior condylar twist angle measured on the preoperative CT scans, while tibial component rotation was determined using the modified range‐of‐motion technique [20]. All patients received routine postoperative care.

### Clinical outcomes

Postoperative clinical outcomes were assessed 1 year after surgery using the Knee Injury and Osteoarthritis Outcome Score (KOOS) [28] as a postoperative patient‐reported outcome measures (PROMs). The KOOS evaluation included five subscales: pain, symptoms, activities of daily living (ADL), sports, and quality of life (QOL). Improvement scores were calculated by subtracting preoperative values from postoperative values for each subscale (∆Pain, ∆Symptom, ∆ADL, ∆sports, and ∆QOL). The knee range of motion was measured preoperatively and postoperatively using a standard clinical goniometer.

### Data analysis

Data analysis was performed using IBM SPSS version 25.0 (IBM Corp., Armonk, NY, USA). Normality was assessed using the Shapiro–Wilk test. Normally distributed data were presented as means with standard deviations (SDs). Comparative analyses of ARA and CRA, mismatch group comparisons, were conducted using the unpaired *t*‐test, Mann–Whitney *U* test, and chi‐square test.

To evaluate the combined effect of ARA and CRA on postoperative outcomes following TKA, we performed a hierarchical cluster analysis using ARA and CRA as clustering variables to identify high‐risk groups for lower outcomes. The unpaired *t*‐test was used to compare postoperative clinical outcomes between subgroups identified in the cluster analysis. Statistical significance was set at *p* < 0.05. A post‐hoc power analysis with an α error of 0.05 revealed a postoperative power (1 − β) of 1.0 for the ARA and CRA comparison and 0.83 for the subgroup comparisons in the cluster analysis, indicating that the sample size was sufficient to detect differences.

### Ethical aspects

The institutional review board of our facility approved the study protocol (no. 2674). Patients and their families were informed that patient data would be used for publication, and written informed consent was obtained.

## RESULTS

**Figure 2:**
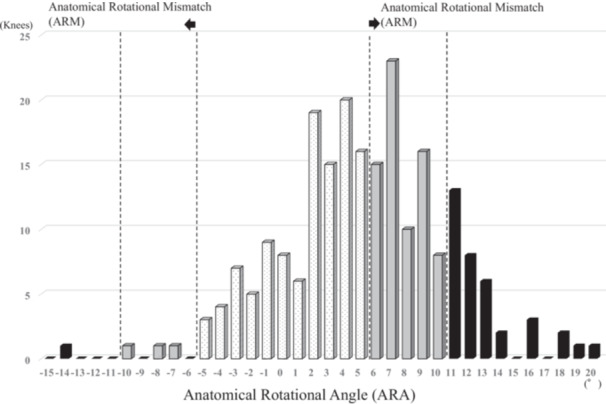
Distribution of postoperative femorotibial anatomical rotational angle (ARA). Internal rotation of the tibia to the femur shows positive value. ARM, anatomical rotational mismatch which was defined as knees with >10°or <−10°, and >5° or <−5° of anatomical rotational angle.

**Figure 3:**
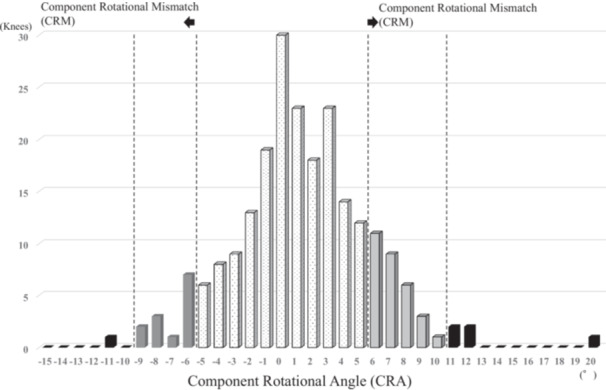
Distribution of postoperative femorotibial component rotational angle (CRA). Internal rotation of the tibial component to the femoral component shows positive value. CRM, component rotational mismatch was defined as knees with >10°or <‐10°, and >5° or <‐5° of component rotational angle.

Table 2 presents postoperative component alignment data in the axial, sagittal, and coronal planes. For femorotibial rotational alignment, a positive value indicates internal rotation of the tibia or tibial component relative to the femur or femoral component in ARA and CRA. Postoperative ARA was 5.2° ± 5.3° (mean ± standard deviation), whereas postoperative CRA was 1.4° ± 4.5°. ARA was significantly larger than CRA in this cohort (*p* < 0.01). The proportion of ARM was 16.5% when defined as ARA over ±10° and 53.1% when defined as ARA over ±5° (Table 2 and Figure 2). The proportion of CRM was 2.8% when defined as CRA over ±10° and 26.3% when defined as CRA over ±5° (Table 2 and Figure 3). The incidence of mismatch was significantly higher in ARM compared with CRM at the ±5° and ±10° thresholds (both *p* < 0.01) (Table 2).

**Table 2:** Postoperative femorotibial rotational angles including the proportion of mismatches and component alignments in computed tomography.

Femorotibital rotational angle	
(tibial internal rotation relative to femur: +)	
Femorotibial Anatomical rotation angle: ARA (°)	5.2 ± 5.3
Proportion of mismatch in ARA (over ±10°)	16.5% (37 knees)
Proportion of mismatch in ARA (over ±5°)	53.1% (119 knees)
Femorotibital Component rotation angle: CRA (°)	1.4 ± 4.5
Proportion of mismatch in CRA (over ±10°)	2.8% (6 knees)
Proportion of mismatch in CRA (over ±5°)	26.3% (59 knees)
Component rotation angle	
Femoral component rotation (°) (Internal rotation: +)	−0.5 ± 2.3
Tibial component rotation (°) (Internal rotation: +)	−4.3 ± 4.6
Component coronal and sagittal angle	
Femoral component coronal angle (°, varus: +)	−0.5 ± 1.3
Femoral component flexion angle (°)	3.5 ± 2.1
Tibial component coronal angle (°, varus: +)	0.3 ± 1.4
Tibial component posterior slope angle (°)	2.7 ± 1.5

Abbreviatios: ARA, anatomical rotational angle between femur and tibia; ARM (10°) and (5°), anatomical rotational mismatch which was defined as knees with >10° or 5° or <‐5° of ARA; CRA, component rotational angle between femoral and tibial components; CRM (10°) and (5°), component rotational mismatch which was defined as knees with >10° or <‐10° and >5° or <‐5° of CRA.

PROMs and knee range of motion significantly improved relative to preoperative values (Table 3) and all KOOS ∆subscales, including ∆Pain, ∆Symptom, ∆ADL, ∆sport and ∆QOL, exceeded the Minimal Clinically Important Change Score (MCID) in KOOS [7]. When comparing knees with and without rotational mismatches in ARA and CRA, knees with ARM showed significantly worse ∆pain subscale scores in KOOS at the ±5° and ±10° thresholds; in contrast, CRM was not associated with significant differences in any KOOS subscale (Table 4).

**Table 3:** Preoperative and postoperative clinical data.

	Preoperative	Postoperative	*p*‐value
KOOS Total	197.1 ± 75.0	378.7 ± 75.3	<0.01*
∆Total (Post‐Pre)	181.4 ± 87.5		
KOOS Pain	44.2 ± 17.5	87.0 ± 12.8	0.02*
∆Pain (Post‐Pre)	43.0 ± 20.3		
KOOS Symptom	51.3 ± 20.9	85.1 ± 12.0	<0.01*
∆Symptoms (Post‐Pre)	34.2 ± 22.1		
KOOS ADL	56.5 ± 16.5	85.0 ± 13.2	<0.01*
∆ADL (Post‐Pre)	29.1 ± 18.3		
KOOS Sports	19.6 ± 17.9	53.5 ± 27.9	<0.01*
∆Sports (Post‐Pre)	34.3 ± 26.0		
KOOS QOL	26.7 ± 17.5	68.0 ± 21.8	<0.01*
∆QOL (Post‐Pre)	41.5 ± 24.2		
Knee Range of motion (°)	110.2 ± 19.1	121.6 ± 15.0	<0.01*
Extension angle (°)	8.4 ± 6.5	0.9 ± 2.2	<0.01*
Flexion angle (°)	118.7 ± 15.4	122.5 ± 14.6	<0.01*

Abbreviations: ADL, activities of daily living; KOOS, Knee injury and Osteoarthritis Outcome Score; QOL, quality of life; *∆*, postoperative value minus preoperative value.

* Significant difference (*p* < 0.05).

**Table 4:** Comparison in clinical outcomes in anatomical and component rotational mismatch.

	ARM (±10°)	non‐ARM (±10°)	*p*‐Value	CRM (±10°)	non‐CRM (±10°)	*p*‐Value
Knees	37	187		6	218	
KOOS ∆Pain	35.7 ± 18.8	44.2 ± 20.1	0.02*	48.1 ± 10.8	42.6 ± 20.3	0.50
KOOS ∆Symptoms	31.3 ± 23.6	34.3 ± 21.2	0.44	44.0 ± 11.4	33.6 ± 21.7	0.24
KOOS ∆ADL	24.6 ± 16.3	29.4 ± 17.6	0.13	31.8 ± 11.1	28.5 ± 17.6	0.64
KOOS ∆Sports	33.9 ± 25.8	34.1 ± 26.0	0.95	48.3 ± 35.2	33.7 ± 25.5	0.17
KOOS ∆QOL	38.0 ± 25.9	42.0 ± 23.9	0.37	44.8 ± 13.3	41.2 ± 24.5	0.72

	**ARM (±5°)**	**non‐ARM (±5°)**	*p*‐Value	**CRM (±5°)**	**non‐CRM (±5°)**	*p*‐Value
Knees	119	105		59	165	
KOOS ∆Pain	40.1 ± 20.3	45.8 ± 19.6	0.03*	38.6 ± 18.7	44.2 ± 20.4	0.07
KOOS ∆Symptoms	33.4 ± 21.6	34.3 ± 21.7	0.76	32.1 ± 19.8	34.5 ± 22.2	0.47
KOOS ∆ADL	26.7 ± 30.7	30.7 ± 17.9	0.08	26.0 ± 17.2	29.5 ± 17.5	0.19
KOOS ∆Sports	34.2 ± 26.2	33.9 ± 25.5	0.93	33.2 ± 26.7	34.4 ± 25.7	0.75
KOOS ∆QOL	39.0 ± 25.0	44.0 ± 23.2	0.12	36.8 ± 22.9	42.9 ± 24.6	0.10

Abbreviations: ADL, activities of daily living; ARM(10°) and (5°), anatomical rotational mismatch which was defined as knees with >10°or 5° or <‐5° of anatomical rotational angle between femur and tibia; CRM(10°) and (5°), component rotational mismatch which was defined as knees with >10° or 5° or <‐5° of component rotational angle between femoral and tibial components; KOOS, Knee injury and Osteoarthritis Outcome Score; QOL, quality of life.

* Significant difference (*p* < 0.05).

**Figure 4:**
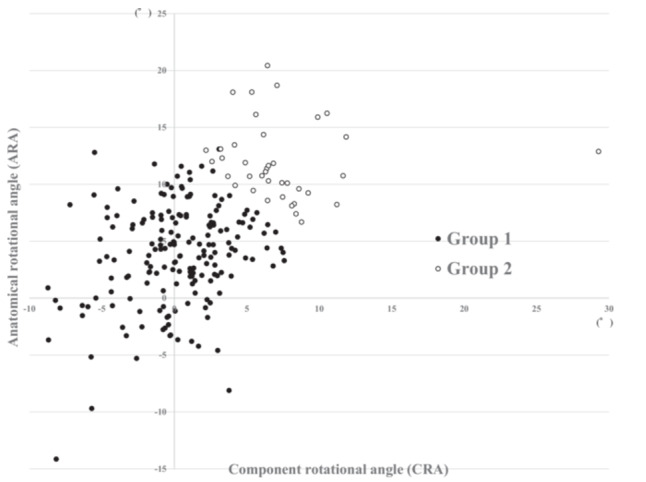
Scatter plot in the cluster analysis based on anatomical rotational angle (ARA) and component rotational angle (CRA). Anatomical rotational angle (ARA) is rotational between femur and tibia, and component rotational angle (CRA) is rotation between femoral and tibial components. Positive value presents an internal rotation of tibial axis relative to the femoral axis in both parameters. The cluster analysis classified knees into two groups Group 1 and Group 2, and Group 2 showed higher ARA and CRA compared to Group 1.

Cluster analysis identified two distinct subgroups: Group 2 (GR2), characterised by higher ARA and CRA (*n* = 39), and Group 1 (GR1), which included knees with low or moderate ARA and CRA values or a high value in only one of the two (*n* = 185; Table 5 and Figure 4). Comparison of clinical outcomes and radiographic data between GR1 and GR2 showed no differences in tibial and femoral component rotational alignments between the groups. However, GR2 exhibited significantly lower ∆pain and ∆ADL subscale scores in KOOS (Table 5).

**Table 5:** Cluster analysis based on both femorotibial anatomical and component rotational angle.

	Group 1	Group 2	*p*‐value
Knees	185	39	
Rotational alignment			
Femorotibial Anatomical rotational alignment (ARA)	3.8 ± 4.5	11.9 ± 3.3	<0.001
(°, Tibial internal rotation relative to femur: +)			
Femorotibial Component rotational alignment (CRA)	0.1 ± 3.5	7.3 ± 4.4	<0.001
(Tibial internal rotation relative to femur: +)			
Tibial component rotational alignment	−4.2 ± 4.5	−5.0 ± 4.9	0.29
(°, Internal rotation relative to Akagi's line: +)			
Femoral component rotational alignment	−0.6 ± 2.3	−0.3 ± 2.4	0.66
(°, Internal rotation relative to SEA: +)			
Preoperative Hip knee ankle angle (°)	170.0 ± 6.2	170.2 ± 5.7	0.84
KOOS ∆Pain	44.4 ± 20.1	34.5 ± 18.5	<0.01*
KOOS ∆Symptoms	34.5 ± 21.2	29.7 ± 23.1	0.21
KOOS ∆ADL	29.5 ± 23.1	23.7 ± 15.4	0.04*
KOOS ∆Sports	33.4 ± 36.9	36.9 ± 27.1	0.46
KOOS ∆QOL	42.3 ± 23.3	35.4 ± 27.5	0.11

Abbreviations: ADL, activities of daily living; KOOS, Knee injury and Osteoarthritis Outcome Score; QOL, quality of life; ∆, postoperative value minus preoperative value.

* Significant difference (*p* < 0.05).

## DISCUSSION

The key findings of this study indicate that both postoperative ARM and CRM occurred, with ARM being more prevalent. Of the two mismatch types, ARM was more closely associated with clinical outcomes after TKA. Furthermore, cluster analysis revealed that patients with concurrent increases in both ARA and CRA experienced poorer clinical outcomes when compared to those with a single type of mismatch.

In this study, when mismatch was defined as ARA exceeding ±10° or ±5°, femorotibial bone‐to‐bone rotational mismatch (ARM) was observed respectively in 16.5% and 53.1% of cases. Only four prior studies have reported on postoperative anatomical femorotibial rotational alignment and ARM in TKA [27, 33, 34, 37]. These studies reported ARM rates of 35.0% and 16.0% for ARA over ±10° [13, 16] and 45.4% and 28.0% for ARA over ±5° [27, 33], which were generally consistent with the present findings. However, the ARM proportion varied slightly depending on the component type and facilities. ARM has often been given less attention than CRM [40], partly because accurate postoperative measurement of the ‘anatomical’ rotational alignment between the femur and tibia requires preoperative and postoperative CT data and specialised evaluation software. Nevertheless, ARM is a common rotational error that warrants attention, and efforts should be made to reduce it through intraoperative techniques. Our ARM shows slightly high proportion of the tibial internal rotation relative to the femur, this may be due to the external tibial component being set to avoid internal rotation. This study further demonstrated that ARM negatively affected patient‐reported pain scores after TKA. This result is consistent with previous reports indicating that excessive ARM between the femur and tibia bones is an independent risk factor for poor functional outcomes after TKA [34]. The lower patient‐reported outcomes with malrotation may be attributed to increased stress on the patellofemoral joint [31] and decreased muscle output [16]. It is plausible that rotational mismatch and alignment changes due to TKA alter patellofemoral joint alignment and muscle positioning. As a result, excessive mechanical stress on the patellofemoral joint due to maltracking, combined with reduced muscle power from misalignment, worsens postoperative clinical outcomes [32].

Regarding femorotibial CRM, when the mismatch was defined as CRA over ±10° or ±5°, CRM occurred in 2.8% and 26.3% of cases, respectively. Previous studies have reported CRM rates of 0%–15% for CRA ± 10° [9, 17, 29, 34] and 14%–48% for CRA ± 5° [1, 23, 33]. More studies have investigated CRM compared with ARM, likely because it is easier to measure postoperative component rotational alignment than to measure postoperative anatomical rotational alignment. However, a wide range of CRM values exist, and proportions vary depending on the component type and facilities, akin to ARM [19, 29, 32, 34]. Tone et al. [33] reported that CRM was observed in 47.5% of bicruciate‐retaining TKAs and 14% of posterior‐stabilised TKAs, emphasising that different component types may yield varying results even under the same measurement conditions. Some surgeons use the range‐of‐motion technique to determine tibial rotational alignment and reduce femorotibial CRM [12, 19]. However, postoperative CRA is influenced by several factors, especially preoperative femorotibial rotational alignment; therefore, achieving perfect femorotibial component rotational alignment (CRA = 0°) with manual or navigation techniques is not always possible. Notably, CRM can be reduced with robotic‐assisted TKA [39]. If robotic technology advances to display intraoperative CRM angles, further reductions in CRM may be achievable. Although this study, along with the findings of Ueyama et al. [34], found no significant clinical impact of CRM on postoperative outcomes, other studies have reported that CRM negatively affects these outcomes [1, 9, 23, 33]. We hypothesise that this discrepancy arose because our study and that of Ueyama et al. had lower CRM rates (around 2%), making it unlikely that low CRM levels would impact postoperative outcomes. Lutzner et al. [25] reported that a CRM exceeding 10° led to altered kinematics and poorer functional outcomes after TKA, and kinematic conflicts relative to natural knee kinematics increased as CRM worsened [26]. Moreover, CRM has been linked to excessive pressure on the patellofemoral joint [36] and TKA revision [40]. Although CRM did not affect outcomes in the current study due to its lower prevalence, it should still be minimised, considering the mechanical and clinical evidence available.

In our cluster analysis, GR2, defined by elevated ARA and CRA, demonstrated poorer postoperative clinical outcomes compared to GR1, which did not exhibit these increased rotational angles. Although previous studies have primarily focused on either CRM or ARM, all TKA procedures inherently involve both anatomical rotational alignment between bones and component rotational alignment between implants. Therefore, it is essential to consider both parameters in postoperative evaluations. Specifically, when assessing postoperative rotation, we must carefully assess whether the TKA exhibits ARM, CRM or both. To the best of our knowledge, this study is the first to highlight the clinical significance of knees with high ARA and CRA. Both this study and that of Ueyama et al. [34] found that ARM had a greater impact on clinical outcomes compared to CRM. However, favourable clinical outcomes can still be observed in some cases despite the presence of ARM. Notably, postoperative anatomical femorotibial rotational alignment is influenced by preoperative anatomical alignment, and ARM may already be present before surgery. Given these findings and previous research, future evaluations of TKA rotational alignment should account for the potential impact of CRM alongside ARM.

The present study has several limitations. First, this research focused on a fixed‐bearing bicruciate substituting prosthesis for knee arthritis with mechanical alignment method, meaning that the findings may not be generalisable to cases involving other types of prostheses, such as cruciate‐retaining or cruciate‐substituting TKAs and to cases involving other types of alignment methods, such as kinematic alignment or personalised alignment. Second, although this study found that only postoperative ARA negatively affected clinical outcomes, CRA may also influenced postoperative clinical outcomes if it had exhibited a wider distribution akin to ARA. Given that we did not intentionally manipulate the CRA range, further studies with a larger sample size are needed to explore this possibility. Third, femorotibial rotational alignment was assessed only in non‐weight‐bearing knee extension and in postoperatively. Other measurements, such as rotational alignment during knee flexion, as well as the changes from extension to flexion or intraoperative change through TKA, may be critical for clinical outcomes and knee kinematics. However, the analysis of these parameters requires a 3D tool, which may be impractical for use in all postoperative patients. Some studies have shown no difference in component mismatch angles under weight‐bearing and non‐weight‐bearing conditions [23]. Fifth, we adopted the ROM technique when tibial rotational alignment was determined, because tibial anatomical landmark seemed unreliable for us. If we had set the tibial component rotational alignment using other methods, such as the anatomical landmark method or the CT‐based navigation method, the results might have been different. Sixth, we set the threshold at ±5° and ±10° based on previous studies, however there is no standard threshold in ARM and CRM. Therefore, we are going to analyse a standard threshold in ARM and CRM with larger sample size. Seventh, we adopted the femoral SEA and tibial Akagi's line as rotational axes on CT scans. These axes are more reliable and commonly used compared to others; however, they may not be ideal for perfectly evaluating anatomical rotational mismatch. Finally, the 1‐year follow‐up period used in this study could be too short to evaluate long‐term clinical outcomes and complications, though there is research indicating that PROMs after TKA show minimal change over a year [6].

## CONCLUSION

Both postoperative ARM and CRM occurred in this study, with ARM being more prevalent following TKA. ARM was more strongly associated with clinical outcomes than CRM in this case series. However, cluster analysis revealed that patients with higher ARA and CRA simultaneously experienced worse clinical outcomes. It is crucial to address both ARM and CRM simultaneously and minimise intraoperatively to optimise clinical outcomes in TKA.

## AUTHOR CONTRIBUTIONS

Kohei Kawaguchi performed the study, carried out the formal analysis and wrote the manuscript. Ryota Yamagami, Kenichi Kono and Hiroshi Inui performed intraoperative data collection. Junfeng Zhang edited the manuscript grammatically. Shuji Taketomi and Sakae Tanaka supervised this study. All authors read and approved the final manuscript.

## CONFLICT OF INTEREST STATEMENT

The authors declare no conflicts of interest.

## ETHICS STATEMENT

This study was prospectively performed at a single institute and retrospectively reviewed. The institutional review board of our facility approved the study protocol (no. 2674). Patients and their families were informed that patient data would be used for publication, and written informed consent was obtained.

## Data Availability

The data that support the findings of the current study are available from the corresponding author (Kohei Kawaguchi) upon reasonable request. The data are not publicly available.

## References

[1] Abdelnasser MK , Elsherif ME , Bakr H , Mahran M , Othman MHM , Khalifa Y . All types of component malrotation affect the early patient‐reported outcome measures after total knee arthroplasty. Knee Surg Relat Res. 2019;31(1):5. 32660572 10.1186/s43019-019-0006-2 PMC7219550

[2] Akagi M , Oh M , Nonaka T , Tsujimoto H , Asano T , Hamanishi C . An anteroposterior axis of the tibia for total knee arthroplasty. Clin Orthop Relat Res. 2004;420:213–219. 10.1097/00003086-200403000-00030 15057100

[3] Beel W , Sappey‐Marinier E , Latifi R , Aït‐Si‐Selmi T , Bonnin MP . Individualised compared to off‐the‐shelf total knee arthroplasty results in lower and less variable patellar tilt. Knee Surg Sports Traumatol Arthrosc. 2024;32(12):3163–3173. 38864165 10.1002/ksa.12234

[4] Bonnin M , Saffarini M , Lustig S , Hirschmann MT . Decoupling the trochlea from the condyles in total knee arthroplasty: the end of a curse? Knee Surg Sports Traumatol Arthrosc. 2024;32(7):1645–1649. 38769816 10.1002/ksa.12267

[5] Bourne RB , Chesworth BM , Davis AM , Mahomed NN , Charron KDJ . Patient satisfaction after total knee arthroplasty. Who is satisfied and who is not? Clin Orthop Relat Res. 2010;468(1):57–63. 19844772 10.1007/s11999-009-1119-9 PMC2795819

[6] Ekhtiari S , Worthy T , Winemaker MJ , de V Beer J , Petruccelli DT , Khanduja V , et al. When does patient function “plateau” after total joint arthroplasty? A cohort study. Int Orthop. 2024;48(9):2283–2291. 39007939 10.1007/s00264-024-06248-8

[7] Emara AK , Pasqualini I , Jin Y , Klika AK , Orr MN , Rullán PJ , et al. Diagnosis‐specific thresholds of the minimal clinically important difference and patient acceptable symptom state for KOOS after total knee arthroplasty. J Bone Jt Surg. 2024;106(9):793–800. 10.2106/JBJS.23.00027 38381811

[8] Ettinger M , Tuecking LR , Savov P , Windhagen H . Higher satisfaction and function scores in restricted kinematic alignment versus mechanical alignment with medial pivot design total knee arthroplasty: a prospective randomised controlled trial. Knee Surg Sports Traumatol Arthrosc. 2024;32(5):1275–1286. 38501253 10.1002/ksa.12143

[9] Fujita M , Matsumoto T , Nakano N , Ishida K , Kuroda Y , Maeda T , et al. Rotational mismatch between femoral and tibial components should be avoided in journey ii bi‐cruciate stabilized total knee arthroplasty. Knee. 2022;38:69–75. 35930895 10.1016/j.knee.2022.07.012

[10] Gunaratne R , Pratt DN , Banda J , Fick DP , Khan RJK , Robertson BW . Patient dissatisfaction following total knee arthroplasty: a systematic review of the literature. J Arthroplasty. 2017;32(12):3854–3860. 28844632 10.1016/j.arth.2017.07.021

[11] Hamilton DF , Lane JV , Gaston P , Patton JT , Macdonald D , Simpson AHRW , et al. What determines patient satisfaction with surgery? A prospective cohort study of 4709 patients following total joint replacement. BMJ Open. 2013;3(4):e002525. 10.1136/bmjopen-2012-002525 PMC3641464 23575998

[12] Ikeuchi M , Yamanaka N , Okanoue Y , Ueta E , Tani T . Determining the rotational alignment of the tibial component at total knee replacement: a comparison of two techniques. J Bone Joint Surg Br. 2007;89(1):45–49. 17259415 10.1302/0301-620X.89B1.17728

[13] Incavo SJ , Wild JJ , Coughlin KM , Beynnon BD . Early revision for component malrotation in total knee arthroplasty. Clin Orthop Relat Res. 2007;458:131–136. 17224835 10.1097/BLO.0b013e3180332d97

[14] Inui H , Yamagami R , Kono K , Kawaguchi K . What are the causes of failure after total knee arthroplasty? J Jt Surg Res. 2023;1(1):32–40.

[15] Ishida K , Shibanuma N , Matsumoto T , Toda A , Oka S , Kodato K , et al. Tibiofemoral rotational alignment affects flexion angles in navigated posterior‐stabilized total knee arthroplasty. Knee Surg Sports Traumatol Arthrosc. 2018;26(5):1532–1539. 28439637 10.1007/s00167-017-4557-z

[16] Jónasson G , Helgason A , Ingvarsson Þ , Kristjánsson AM , Briem K . The effect of tibial rotation on the contribution of medial and lateral hamstrings during isometric knee flexion. Sports Health. 2016;8(2):161–166. 26721286 10.1177/1941738115625039 PMC4789934

[17] Kawaguchi K , Inui H , Taketomi S , Yamagami R , Kono K , Sameshima S , et al. Preoperative tibiofemoral rotational alignment is a risk factor for component rotational mismatch in total knee arthroplasty. Knee. 2021;29:448–456. 33743260 10.1016/j.knee.2021.02.028

[18] Kawaguchi K , Inui H , Yamagami R , Kono K , Kage T , Muramakami R , et al. Effect of component rotational alignment on femorotibial rotational alignment in total knee arthroplasty: comparison between mobile and fixed bearing. J Jt Surg Res. 2023;1(1):86–91.

[19] Kawaguchi K , Yamagami R , Kenichi K , Kage T , Murakami R , Arakawa T , et al. Intraoperative reliability of the tibial anteroposterior axis “akagi's line” in total knee arthroplasty. J Exp Orthop. 2024;11(2):e12020. 38617135 10.1002/jeo2.12020 PMC11009861

[20] Kawaguchi K , Inui H , Yamagami R , Kenichi K , Sameshima S , Kage T , et al. A new technique for determining the rotational alignment of the tibial component during total knee arthroplasty. Knee. 2021;29:323–331. 33684863 10.1016/j.knee.2021.02.006

[21] Keshmiri A , Maderbacher G , Baier C , Zeman F , Grifka J , Springorum HR . Significant influence of rotational limb alignment parameters on patellar kinematics: an in vitro study. Knee Surg Sports Traumatol Arthrosc. 2016;24(8):2407–2414. 25399346 10.1007/s00167-014-3434-2

[22] Klem NR , Smith A , O'Sullivan P , Dowsey MM , Schütze R , Kent P , et al. What influences patient satisfaction after tka? A qualitative investigation. Clin Orthop Relat Res. 2020;478(8):1850–1866. 32732567 10.1097/CORR.0000000000001284 PMC7371044

[23] Kokubu Y , Kawahara S , Mizu‐Uchi H , Hamai S , Akasaki Y , Sato T , et al. Component rotational mismatch in the standing position is a potential risk factor for unfavourable functional outcomes after total knee arthroplasty. J Exp Orthop. 2024;11(3):e12069. 38957227 10.1002/jeo2.12069 PMC11217670

[24] Koutserimpas C , Saffarini M , Bonnin M , Hirschmann MT , Lustig S . Optimizing the patellofemoral compartment in total knee arthroplasty: Is it time for dynamic assessment? Knee Surg Sports Traumatol Arthrosc. 2025;33(2):387–392. 39224026 10.1002/ksa.12450

[25] Lützner J , Kirschner S , Günther KP , Harman MK . Patients with no functional improvement after total knee arthroplasty show different kinematics. Int Orthop. 2012;36(9):1841–1847. 22643798 10.1007/s00264-012-1584-8 PMC3427439

[26] Maderbacher G , Keshmiri A , Springorum HR , Maderbacher H , Grifka J , Baier C . Influence of component rotation in total knee arthroplasty on tibiofemoral kinematics‐a cadaveric investigation. J Arthroplasty. 2017;32(9):2869–2877. 28434698 10.1016/j.arth.2017.03.055

[27] Mochizuki T , Sato T , Tanifuji O , Watanabe S , Kobayashi K , Endo N . Extrinsic factors as component positions to bone and intrinsic factors affecting postoperative rotational limb alignment in total knee arthroplasty. J Arthroplasty. 2018;33(7):2100–2110. 29506933 10.1016/j.arth.2018.02.009

[28] Nakamura N , Takeuchi R , Ishikawa H , Saito T , Sawaguchi T , Goldhahn S . Cross‐cultural adaptation and validation of the japanese knee injury and osteoarthritis outcome score (KOOS). J Orthop Sci. 2011;16(5):516–523. 21766211 10.1007/s00776-011-0112-9

[29] Nedopil AJ , Howell SM , Rudert M , Roth J , Hull ML . How frequent is rotational mismatch within 0°±10° in kinematically aligned total knee arthroplasty? Orthopedics. 2013;36(12):e1515–e1520. 24579223 10.3928/01477447-20131120-15

[30] Nicoll D , Rowley DI . Internal rotational error of the tibial component is a major cause of pain after total knee replacement. J Bone Joint Surg Br. 2010;92(9):1238–1244. 20798441 10.1302/0301-620X.92B9.23516

[31] Steinbrück A , Schröder C , Woiczinski M , Schmidutz F , Müller PE , Jansson V , et al. Mediolateral femoral component position in tka significantly alters patella shift and femoral roll‐back. Knee Surg Sports Traumatol Arthrosc. 2017;25(11):3561–3568. 28681088 10.1007/s00167-017-4633-4

[32] Talbot S , Zordan R , Sasanelli F , Sun M . Preoperative quadriceps malalignment is associated with poor outcomes after knee replacement which are avoided by external rotation of the femoral component. Knee Surg Sports Traumatol Arthrosc. 2025;33(4):1418–1427. 39666776 10.1002/ksa.12544 PMC11948163

[33] Tone S , Hasegawa M , Naito Y , Wakabayashi H , Sudo A . Association between pre‐ and postoperative rotational mismatches of the femorotibial components and bones in bi‐cruciate retaining and posterior stabilized total knee arthroplasty. Sci Rep. 2023;13(1):14902. 37689778 10.1038/s41598-023-42243-6 PMC10492851

[34] Ueyama H , Minoda Y , Sugama R , Ohta Y , Yamamura K , Nakamura S , et al. Malrotation of the fixed‐bearing posterior stabilized total knee prosthesis causes a postoperative rotational mismatch between the femur and tibia. Knee Surg Sports Traumatol Arthrosc. 2020;28(12):3810–3820. 31996931 10.1007/s00167-020-05864-2

[35] Ueyama H , Minoda Y , Sugama R , Ohta Y , Takemura S , Nakamura H . Mobile‐bearing prosthesis suppresses the postoperative rotational mismatch and improves patient‐reported outcome measurements better than fixed‐bearing prosthesis: Rotational analysis by 3d measurement in total knee arthroplasty. Arch Orthop Trauma Surg. 2023;143(11):6781–6790. 37418005 10.1007/s00402-023-04971-2

[36] Verlinden C , Uvin P , Labey L , Luyckx JP , Bellemans J , Vandenneucker H . The influence of malrotation of the femoral component in total knee replacement on the mechanics of patellofemoral contact during gait: An in vitro biomechanical study. J Bone Joint Surg Br. 2010;92(9):737–742. 20436014 10.1302/0301-620X.92B5.22603

[37] Watanabe S , Sato T , Omori G , Koga Y , Endo N . Change in tibiofemoral rotational alignment during total knee arthroplasty. J Orthop Sci. 2014;19(4):571–578. 24817492 10.1007/s00776-014-0565-8

[38] Wu G , Cao Y , Song G , Li Y , Zheng T , Zhang H , et al. The increased tibiofemoral rotation: a potential contributing factor for patellar maltracking in patients with recurrent patellar dislocation. Orthop Surg. 2022;14(7):1469–1475. 35698275 10.1111/os.13358 PMC9251321

[39] Yamamoto A , Kaneko T , Takada K , Yoshizawa S . Robotic‐assisted total knee arthroplasty improves the rotational mismatch between femoral and tibial components, but not the forgotten joint score 12: a single‐center retrospective cohort study. J Exp Orthop. 2023;10(1):133. 38062307 10.1186/s40634-023-00705-w PMC10703751

[40] Young SW , Saffi M , Spangehl MJ , Clarke HD . Unexplained pain following total knee arthroplasty: is rotational malalignment the problem? Knee. 2018;25(2):329–334. 29526396 10.1016/j.knee.2018.01.011

